# King Edward's Hospital Fund for London

**Published:** 1923-01

**Authors:** 


### KING EDWARD'S HOSPITAL FUND FOR LONDON.

### THE STATISTICAL REPORT FOR 1921.

SOME time ago the Council of King Edward's Hospital Fund asked for suggestions for the improvement of the annual statistical report. We do not know if the whole of the responses have been considered, but the report just published does not materially differ from its predecessors.

There is, however, one new, important and most interesting feature. It will be remembered that the report mainly dealt with an analysis of the expenditure of the voluntary hospitals of London. To this is now added for the first time an analysis of the income of these institutions.

The report deals with 113 hospitals with 10,340 beds in daily occupation during 1921. To these beds were admitted 163,438 patients. The out-patient departments received 1,451,538 out-patients, who made 6,160,224 attendances. These figures, impressive though they are, signify only the results of the work. The by-products, if we may use the expression, are also remarkable. In addition to all that was done for the patients and their families, while this great work was going on, in conjunction with it were trained a large number of the medical men of the future, of the nurses that one day or other will enter and stay awhile in the houses of most of us, of masseuses, workers on the social-welfare side, and miscellaneous details of the great army of healing.

In money the cost for the year of the 113 hospitals, including everything except capital expenditure, was £2,776,603. The corresponding figure in 1913 was £1,192,199, and in 1920 £2,784,277. Thus it will be seen that, despite the falling cost of living, the hospitals collectively spent as much last year as the year before. When the expenditure is analysed it is found that there is generally a decrease under all headings except Domestic (heating, lighting, laundry, and the like), and Salaries and Wages, which have again increased considerably.

From the above it will be gathered that the hospitals had to provide, as compared with 1913, an additional amount of £1,584,404, representing an increased expenditure of nearly 133 per cent. over 1913. The total income rose from £1,470,282 in 1913 to £2,566,114 in 1921, an increase of nearly 75 per cent. The last total does not include grants from the Voluntary Hospitals Commission. Under every heading there is an augmentation, people are giving more to hospitals, and the patients are paying substantially towards their cost of maintenance. The difficulty lies in the fact that the increase in expenditure has outpaced the increase in income.

What are patients doing to help the hospitals in their financial anxiety? They provided £78,000 in 1913 and £420,000 in 1921. Moreover £306,000 was received in the latter year for work done for Public Authorities, as against £11,000 in 1913. When the balance is struck in individual cases it is found that last year 48 hospitals had surpluses, and 65 had deficits, the aggregate net deficiency being £210,489. This is an improvement upon 1920, when the deficit was £382,965. It is interesting to find for the first time in this report material for dividing the aggregate income of Metropolitan hospitals under headings. Thus we discover the percentage of the total General Fund income is 18.3 under investments, 39.4 under voluntary gifts, 28.5 under earnings (patients' payments, Public Authorities, &c.), and 13.8 under extraordinary income—mostly legacies. These are aggregates, but the same system is applied to individual hospitals

No changes have been made in the form of the tables which deal with the cost of working, and those using the report are warned that deductions as to extravagance or parsimony are not to be made from the comparisons. The main object, easily realisable, is to assist managers to avoid as far as possible increases in cost which are not represented by an increase in the quantity or quality of the work done. This is quite reasonable, but all the same some of the comparisons inevitably lead to the examination side by side of hospital with hospital and group with group. Arising from such observation one must ask, for instance, why is the hospital with the medical school so much more expensive than the non-teaching hospital ? The twelve teaching hospitals spend on an average £196 to maintain an occupied bed, while the large general hospital, without schools, can give the same service for £157. Theoretically the teaching hospital should be cheaper to maintain in view of the large amount of unpaid service given by the students, but in point of fact the most cheaply run teaching hospital is as expensive as the average of all the larger non-teaching hospitals. Let us take the in-patient expenditure of two hospitals with an average bed occupation of about 200 in each case. A has a school, B has not. A spends an average of £197 on each bed, B manages with £134. A spends £80 per bed on salaries and wages, B spends under £50. Each is a hospital of the highest reputation not likely to be either extravagant or stingy. What is the essential difference (taking it that the cost of the medical school has been taken out of A's figures) which leads to such a remarkable variation in cost ?

We warmly congratulate Mr. H. R. Maynard, the secretary of King Edward's Hospital Fund, on the helpful tables he has compiled, and upon the lucid explanations and summaries that accompany them.

### THE COMBINED APPEAL AND THE FUTURE.

THE meeting of King Edward's Hospital Fund for London, over which the Prince of Wales presided at St. James' Palace, had unusual interest this year from its coincidence with the conclusion of the combined appeal. The Fund's annual distribution was again £220,000, and the recent munificent legacies from Lord Mount Stephen and Sir Thomas Sutherland have helped to replenish the capital fund which was depleted by the great emergency grants distributed during the financial crisis that succeeded the war. Sir Alan Anderson, Chairman of the Revenue Committee, said that the Director-General estimated the grand total of the Combined Appeal Fund to be £435,000. They hoped that the advertisement prize fund and the educational auxiliary under Lord Burnham's chairmanship, would raise the balance. The general appeal closes on December 31.

The Revenue Committee is now considering permanent methods of increasing the income of hospitals. One is the organisation of support from domestic workers, to form, it is hoped, a permanent branch of the League of Mercy. The second is the organisation of contributions from wage-earners generally. The propaganda, Sir Alan continued, for the Combined Appeal had emphasised the hospitals' policy and programme for those who became regular contributors, and the need for the latter's organisation. To this, he felt sure, a large part of the success of the Combined Appeal was due.

**The Prince on the Provincial Hospitals.**—In relation to this the Prince of Wales' speech contained a passage worth quoting. The programme outlined in the report of Lord Cave's Committee is based, said the Prince, largely on two things :—

" One is the success of many of the provincial hospitals in organising workshop contributions. The other is the growth of the King's Fund from a mere collecting and distributing agency into a central administrative body, which, being itself voluntary respects the independence of the voluntary hospitals."

It is timely that the Prince of Wales should declare, and the King's Fund record, that the Provincial Hospitals have set an example to London in this matter, and were the first to lead the way toward the new policy by which the voluntary system seeks to live. The provincial hospitals may well be proud of this tribute, which, as our pages have for long borne witness, has been thoroughly earned. We hope, however, that the second point made by the Prince in this passage will not be lost upon them. If the London hospitals have learnt with advantage from the provinces in the matter of organised contributions, the provincial hospitals may learn from London of the value of a central fund. A King's Fund for the Provincial Hospitals has often been mooted. A fit equivalent has never been achieved. Perhaps the ultimate success of the attempts toward co-ordination now proceeding under the Local Committees can be judged by the eventual success or failure of such a scheme. We hope that the suggestions contained in the Prince of Wales' speech will not be lost upon hospital men in the provinces.

A Bible has been presented to the Chapel of King's College Hospital in memory of Margaret Flora Victoria Bull, who had left her savings to the hospital. The girl's interest was aroused through her mother's activity for the Hospital Ladies' Association.

The International Labour Review, the monthly publication of the International Labour Office of the League of Nations, publishes some interesting notes on Industrial Hygiene. These notes refer to such subjects as : Health Service in Industry ; Public Health in Great Britain ; Development of Industrial Hygiene in the United States ; Progress in Ventilation Standards in the United States ; Industrial Hygiene in the Chemical Industry ; Influence of Industry on Alcoholism ; Tetrachlorethane Poisoning in Industry ; Dermatosis caused by Cutting Oils and Lubricating Compounds ; Experimental Production of Cancer by Tar.

The Review is issued from the London Office, 26 Buckingham Gate, S.W.1.

